# Neural adaptations to temporal cues degradation in early blind: insights from envelope and fine structure vocoding

**DOI:** 10.3389/fnins.2025.1493641

**Published:** 2025-05-02

**Authors:** Hyo Jung Choi, Jeong-Sug Kyong, Jong Ho Won, Hyun Joon Shim

**Affiliations:** ^1^Department of Otorhinolaryngology-Head and Neck Surgery, Nowon Eulji Medical Center, Eulji University School of Medicine, Seoul, Republic of Korea; ^2^Eulji Tinnitus and Hearing Research Institute, Nowon Eulji Medical Center, Seoul, Republic of Korea; ^3^Sensory Organ Institute, Medical Research Institute, Seoul National University, Seoul, Republic of Korea; ^4^Department of Radiology, Konkuk University Medical Center, Seoul, Republic of Korea; ^5^Alston & Bird, LLP, Washington, DC, United States

**Keywords:** speech intelligibility, temporal degradation, vocoder, temporal envelope, temporal fine structure, N2 and P3b

## Abstract

In our previous study, early-blind individuals have better speech recognition than sighted individuals, even when the spectral cue was degraded using noise-vocoders. Therefore, this study investigated the impact of temporal envelope degradation and temporal fine structure (TFS) degradation on vocoded speech recognition and cortical auditory response in early blind individuals compared to sighted individuals. The study included 20 early-blind subjects (31.20 ± 42.5 years, M: F = 11:9), and 20 age- and -sex-matched sighted subjects. Monosyllabic words were processed using the Hilbert transform to separate the envelope and TFS, generating vocoders that included only one of these components. The amplitude modulation (AM) vocoder, which contained only the envelope component, had the low-pass filter's cutoff frequency for AM extraction set at 16, 50, and 500 Hz to control the amount of AM cue. The frequency modulation (FM) vocoders, which contained only the TFS component, were adjusted to include FM cues at 50%, 75%, and 100% by modulating the noise level. A two-way repeated measures ANOVA revealed that early-blind subjects outperforming sighted subjects across almost all AM or FM-vocoded conditions (*p* < 0.01). Speech recognition in early-blind subjects declined more with increasing TFS degradation, as evidenced by a significant interaction between group and the degree of TFS degradation (*p* = 0.016). We also analyzed neural responses based on the semantic oddball paradigm using the N2 and P3b components, which occur 200–300 ms and 250–800 ms after stimulus onset, respectively. Significant correlations were observed between N2 and P3b amplitude/latency and behavioral accuracy (*p* < 0.05). This suggests that early-blind subjects may develop enhanced neural processing strategies for temporal cues. In particular, preserving TFS cues is considered important for the auditory rehabilitation of individuals with visual or auditory impairments.

## 1 Introduction

Auditory temporal resolution refers to the auditory system's ability to detect and process rapid changes in sound over time. Temporal processing allows the auditory system to extract important features, such as pitch, timing, and the rhythmic structure of speech, which are crucial for distinguishing between different speech sounds and understanding speech, especially in noisy environments (Haggard, 1984; McKay et al., 2013; McFarlane and Sanchez, 2024). An acoustic signal in the temporal domain is decomposed into a slowly varying temporal envelope and a rapidly varying temporal fine structure (TFS) (Hilbert, 1912). The temporal envelope cue plays a crucial role in speech recognition in quiet environments environments (Drullman et al., 1994; Shannon et al., 1995). Studies using a noise vocoder, where the bandwidth is divided and the temporal envelope information of each band is preserved, have shown that even when most of the spectral cue of speech is removed, 90% of words were correctly identified through the temporal envelope (Shannon et al., 1995). Smith et al. found that when using 4–16 frequency bands of an “auditory chimera,” in which the envelope from one sound is paired with the TFS of another, the recognition of English speech was dominated by the envelope (Smith et al., 2002), whereas the recognition of tonal languages, such as Mandarin Chinese, relies more on TFS (Xu and Pfingst, 2003; Wang et al., 2015). TFS bcomes important in sound localization (Yin and Chan, 1990; Smith et al., 2002; Borjigin et al., 2022), as well as pitch perception through fundamental-frequency (Moore, 1973; Houtsma and Smurzynski, 1990; Qin and Oxenham, 2005) and music perception (Smith et al., 2002; Heng et al., 2011). However, there has been a long-standing debate on whether TFS contributes to masking release through spatial cues and F0 information (Lorenzi et al., 2006; Moore, 2008; Oxenham, 2008; Gnansia et al., 2009; Oxenham and Simonson, 2009). A recent study found that greater TFS sensitivity does not enhance masking release from F0 or spatial cues but aids resilience to reverberation and reduces listening effort, as indicated by faster response times (Borjigin and Bharadwaj, 2025).

But blind individuals rely solely on auditory signals for communication, making it essential to investigate their speech perception abilities in comparison with sighted individuals. This is particularly important for developing rehabilitation programs for visually impaired individuals. Early-blind individuals, who were either blind at birth or became blind within the first year of life, experience compensatory mechanisms in the brain that enhance the processing of non-visual senses such as hearing and touch. This enhancement also extends to their auditory temporal resolution abilities. Several studies have demonstrated that early-blind individuals show advantages in temporal-order judgment ability (Weaver and Stevens, 2006), temporal patterns (Bae et al., 2022), auditory temporal resolution (Muchnik et al., 1991), temporal modulation detection (Shim et al., 2019), and temporal attention for stimulus selection (Röder et al., 2007) over sighted subjects.

Our previous study demonstrated that speech recognition declined as spectral cues were reduced (i.e., with a decreased number of channels) in both blind and sighted individuals. However, early-blind individuals have better speech recognition than sighted individuals, even when the spectral cue was degraded using noise-vocoders with different numbers of channels (Choi et al., 2024a). Nontheless, spectral degradation had a greater impact on speech recognition with increasing degradation in early-blind subjects. Therefore, this study focused on temporal resolution to determine whether early-blind subjects have speech recognition advantages over sighted subjects in environments with various levels of degraded temporal resolution. Blind individuals are strongly reliant on auditory cues for communication without visual cues, which could markedly disrupt their daily life, even with minor impairments in temporal cues. However, few studies have examined speech recognition in blind individuals in the context of limited auditory temporal cues.

To investigate the impact of temporal resolution degradation on the speech recognition of early-blind individuals, we used noise-vocoded speech. Monosyllabic words were processed using the Hilbert transform to separate the envelope and TFS, generating vocoders that included only one of these components. Using an amplitude modulation (AM) vocoder, which contained only the envelope component, the low-pass filter's cutoff frequency for AM extraction was set at 16, 50, and 500 Hz to control the envelop cut-off frequency of the AM cue (Shannon et al., 1995). The frequency modulation (FM) vocoders, which contained only the TFS component, were adjusted to include FM cues at amount of 50%, 75%, and 100% by modulating the noise level (Moon et al., 2014).

In addition, we used the “semantic oddball paradigm” to investigate the neural correspondence of speech recognition affected by degradation of the temporal cues in early-blind individuals. We focused on the N2 and P3b components, which are associated with higher-order neural processing for stimulus discrimination and evaluation (Voola et al., 2023). These components likely depend more on top-down processing when temporal speech cues are degraded. The N2 component is a negative deflection starting around 200–300 ms post-stimulus (Folstein and Van Petten, 2008), and is a sensitive index for examining the course of semantic and phonological encoding (Schmitt et al., 2000) or listening to sound with the oddball paradigm (Finke et al., 2016; Voola et al., 2023). P3b, which occurs between 250 and 800 ms, exhibits a variable peak that is dependent on the individual's response, and its amplitudes are typically greater over the parietal electrodes. P3b was measured using the parietal electrodes (CP1, CP2, P3, P4, and Pz), as outlined in Finke et al. (2016). P3b is associated with the judgment of stimulus inconsistency while updating working memory. Prolonged latencies may represent slower stimulus evaluation (Beynon et al., 2005; Henkin et al., 2015). Our previous study using a one-syllable oddball paradigm with animal and non-animal stimuli across varying channel vocoder conditions confirmed that the N2 and P3b responses reflect cortical effects. This indicates that semantic integration is less efficient due to reduced spectral information in speech (Choi et al., 2024b). Therefore, we assessed semantic processing, as represented by the N2 and P3b responses, using the same paradigm with degradation of the envelope and TFS cues, and compared these responses between early-blind and sighted subjects.

## 2 Subjects and methods

### 2.1 Subjects

The study population included a group of 20 early-blind subjects (31.20 ± 4.25 years, male: female [M: F] = 11:9) and a control group of 20 sighted subjects (28 ± 6.9 years, male: female [M: F] = 11:9). There was no significant difference in age between the two groups (*p* < 0.05). All of the subjects were right-handed, aged <40 years, and had normal hearing thresholds in both ears (≤20 dB hearing level at 0.25, 0.5, 1, 2, 3, 4, and 8 kHz). They had no other neurological or ontological problems. The early-blind group only included people who were blind at birth or who became blind within 1 year of birth, and were classified in categories 4 and 5 according to the 2006 World Health Organization guidelines for the clinical diagnosis of visual impairment (category 4, “light perception” but no perception of “hand motion”; category 5, “no light perception”). Table 1 provides the characteristics of the blind subjects. The study was conducted by the Declaration of Helsinki and the recommendations of the Institutional Review Board of Nowon Eulji Medical Center, with written informed consent from all subjects. Informed consent was obtained verbally from the blind subjects in the presence of a guardian or third party. The subjects then signed the consent form, and a copy was given to them.

**Table 1:** Clinical characteristics for the early-blind subjects.

**Subject**	**Age (yeas)**	**Onset**	**Sex**	**Visual acuity**	**Cause of blindness**
B01	26	Birth	M	No light perception	Corneal opacity
B02	29	Birth	F	No light perception	Retinopathy of prematurity
B03	25	Birth	M	NO light perception	Cause unknown
B04	39	Birth	M	Light perception	Optic atrophy
B05	26	Birth	M	Light perception	Xanthochromism
B06	25	Birth	M	No light perception	Optic atrophy
B07	27	Birth	M	No light perception	Retinopathy of prematurity
B08	31	Birth	F	No light perception	Retinopathy of prematurity
B09	37	Birth	F	No light perception	Optic atrophy
B10	30	Birth	F	No light perception	Glaucoma
B11	31	Birth	F	No light perception	Retinopathy of prematurity
B12	34	Birth	F	Light perception	Retinoblastoma
B13	31	Birth	M	Light perception	Cause unknown
B14	36	Birth	F	Light perception	Retinopathy of prematurity
B15	28	Birth	F	Light perception	Optic atrophy
B16	31	Birth	F	No light perception	Poor eye development
B17	32	Birth	M	No light perception	Persistent hyperplastic primary vitreous
B18	28	Birth	M	No light perception	Retinopathy of prematurity
B19	27	Birth	M	Light perception	Microphthalmos
B20	37	Birth	M	No light perception	Retinopathy of prematurity

### 2.2 AM- and FM-vocoded speech

Stimuli were recorded by a male speaker reading five lists of 25 Korean monosyllabic words in a soundproof booth using a lapel microphone (BY-WMA4 PRO K3; BOYA, Shenzhen, Hong Kong). All the recorded stimuli were sampled at a rate of 44,100 Hz. The overall root mean square amplitude was normalized to −25 dB relative to full scale using Adobe Audition (Adobe Systems, San Jose, CA, USA), ensuring that the average signal intensity was 25 dB below the maximum possible digital level to maintain consistent stimulus intensity across recordings.

For the amplitude modulation vocoder, the input signal was first filtered into eight frequency bands ranging from 80 to 8,000 Hz, with each band equally spaced on an equivalent rectangular bandwidth scale (Glasberg and Moore, 1990).

**Figure 1:**
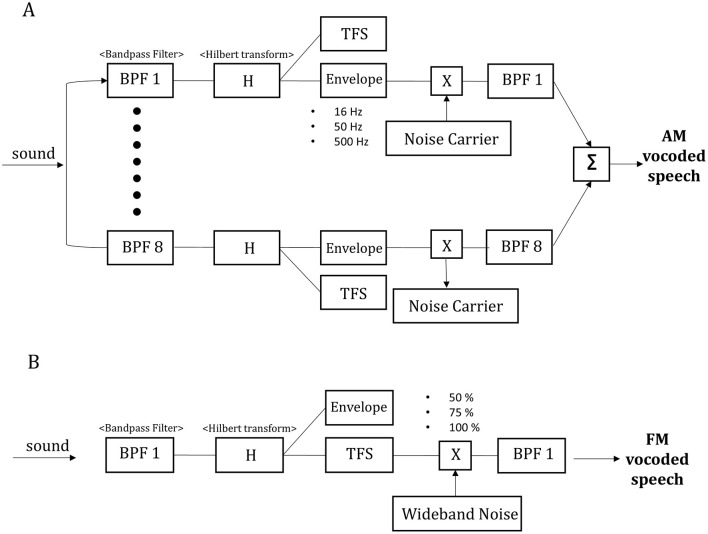
Schematic diagram of the amplitude modulation (AM) vocoder **(A)**. The input sounds were divided into eight channel bands using bandpass filters (BPF1 to BPF8), and each filtered sound was subjected to Hilbert transformation (H) to extract the envelope of each band, removing the temporal fine structure (TFS). The temporal envelope cutoff frequencies for AM extraction were set at 16, 50, and 500 Hz. The vocoded speech signal was generated by adding a noise carrier to the envelopes in each channel band. Finally, the signals were passed through each bandpass filter and summed to produce the AM-vocoded speech sound. Schematic diagram of the frequency modulation (FM) vocoder **(B)**. The input sound was passed through a single-frequency bandpass filter (BPF1) and the filtered sound was subjected to H to extract the TFS. The amount of TFS was manipulated by wideband noise (50, 75, and 100%).

The band cutoffs were determined to ensure that the filter bandwidths closely matched those of the auditory filters. The cutoff frequencies of each bandpass filter were determined using a logarithmically spaced frequency range based on the Greenwood function (80, 214, 424, 748, 1,250, 3,234, 5,103, and 8,000 Hz). The cutoff frequency of the low-pass filter for temporal envelope extraction was applied at 16, 50, and 500 Hz. The central frequency of each channel was calculated as the geometric mean between the two corresponding cutoff frequencies associated with that specific channel. The amplitude envelope for each frequency band was then extracted through Hilbert transform. Finally, we summed the sub-band signals to generate the noise-vocoded signals (Shannon et al., 1995; Faulkner et al., 2012; Evans et al., 2014) (Figure 1A).

**Figure 2:**
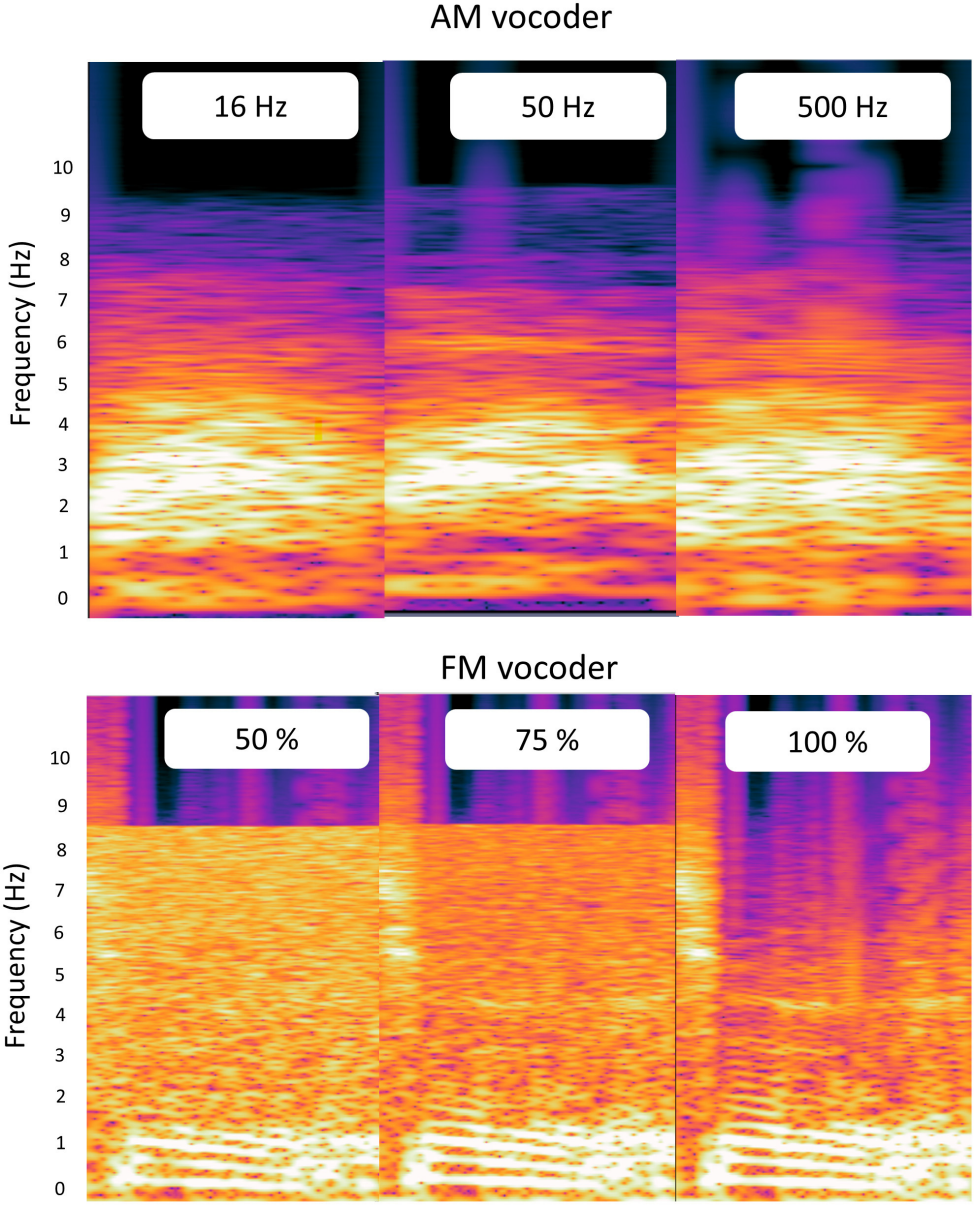
Spectrograms of the amplitude modulated (AM) and frequency modulated (FM) vocoder outputs for the word “MAL”. The top row shows the spectrograms for the AM vocoder at three different temporal envelope cutoff frequencies (16, 50, and 500 Hz). The bottom row displays the spectrograms for the FM vocoder at three different amounts of temporal fine structure (TFS; 50, 75, and 100%).

For the FM-vocoder, the input signal was first filtered using a wideband bandpass filter (80–8,000 Hz; Figure 1B). The Hilbert transform was then applied to each subband signal to decompose it into its analytic signal, from which the envelope and temporal fine structure were extracted. The TFS component was isolated by retaining only the phase information, represented by the cosine value of the phase of the analytic signal. A separate set of band-limited noise signals was generated and filtered using the same wideband bandpass filter as employed for the input signal. The root mean square of band-limited noise signals was set to that of analytic signal. To vary the amount of FM cues available in the output signals, we used the phase randomization technique of Moon et al. (2014).


(1) $$\begin{array}{r} Y\left(t\right)=abs\left(X\left(t\right)\right)\times \cos(angle(\left[\left(1-NF\right)\times X\left(t\right)\right]\\ +\left[NF\times N\left(t\right)\right]))) \end{array}$$

where Y (t) is the output stimulus, X(t) is the analytic signal, N(t) is the filtered noise in an analytic form, and NF is a “noise factor” from 0 to 1. We added the weighted random noise component (i.e., analytic signal [NF × N(t)]) to the weighted original analytic signal [(1 – NF) × X(t)]. Then, the randomized TFS was obtained by taking the cosine value of the angle of these mixed signals. The randomized TFS was then modulated with the envelope of the 1-band signal. We tested NF values of 0.5, 0.25, and 0. The NF value of 0.5 produced the output signal containing 50% of the FM cues for the original signal. The NF value of 0.25 produced the output signal including 75% of the original FM cues. Finally, the NF value of 0 preserved the intact (100%) FM cues. Vocoding was performed using a custom MATLAB script (2020a, Mathworks, Inc., Natick, MA, USA), in which the spectra became more blurred as the cut-off frequency of the envelope decreased and as the preserved amount of the TFS decreased, as shown in Figure 2.

### 2.3 Procedures

#### 2.3.1 Behavioral test

Speech recognition using the AM and FM vocoders was compared between early-blind and sighted subjects. The perception of one-syllable words was tested under three different amounts of envelope cues (AM vocoder: 16, 50, and 500 Hz cutoff frequency) and three different amounts of TFS cues (FM vocoder: 50, 75, and 100%) using five lists, each containing 25 Korean monosyllabic words. The participants were asked to repeat the words after they were presented through a loudspeaker placed 1 meter in front of the subject's ear. All tests were conducted in a soundproof room with an audiometer (Madsen Astera 2; GN Otometrics, Taastrup, Denmark), and the stimuli was presented at 70 dB SPL. The word recognition scores were calculated as the percentage of correctly repeated words.

#### 2.3.2 N2 and P3b

The neural response was recorded across 31 AG-Ag/Cl sintered electrodes placed according to the international 10-20 system (Klem, 1999) and referenced at FCz in an elastic 32-channel cap using the actiCHamp Brain Products recording system (BrainVision Recorder Professional, V.1.23.0001, Brain Products GmbH, Munich, Germany). All recordings were made in a dimly lit, sound-attenuated, electrically shielded chamber. The electro-oculogram (EOG) and electrocardiogram (ECG) were tagged to trace the subject's eye movement and heartbeat, respectively. The electroencephalogram (EEG) data were digitized online at a sampling rate of 1,000 Hz. The ground electrode was placed between electrodes Fp1 and Fp2. Software filters were set at low (0.5 Hz) and high (70 Hz) cutoffs. A notch filter at 60 Hz was set to prevent powerline noise, and the impedances of all scalp electrodes were kept below 5 kΩ using EEG electrode gel throughout the recording, following the manufacturer's instructions.

##### 2.3.2.1 Oddball paradigm

Based on the semantic oddball paradigm, the subjects listened to animal stimuli or non-animal but meaningful stimuli (Choi et al., 2024b). Overall, 70% of the trials involved animal words (e.g., mouse, snake, bear; all monosyllable in Korean). The remaining 30% consisted of monosyllable non-animal words but belonged to a different semantic category. The subjects sat comfortably in the soundproof booth and listened to the animal or non-animal words in a random order. The researchers told the subjects to expect to hear an animal word and instructed them to press the button as quickly and accurately as possible upon hearing the word. In the cutoff frequency condition (16, 50, and 500 Hz) and the TFS condition (50, 75, and 100%), 210 animal words and 90 non-animal words were presented in six blocks, and the subjects listened to a total of 900 trials in each condition. The inter-stimulus interval was fixed at 2,000 ms, and a jitter of 2–5 ms was allowed. The order of presentation was randomized within the blocks and the order of blocks was counterbalanced among subjects using E-Prime software (version 3, Psychology Software Tools, Sharpsburg, PA). Each subject had a 5-min break after completing each block. The subjects had a familiarizing session before starting the trials to ensure that they understood the task and that their muscles were relaxed. The intensity of sound was fixed at 70 dB SPL when calibrated at the listener's head position, 1 m from the loudspeaker.

##### 2.3.2.2 Data processing

**Figure 3:**
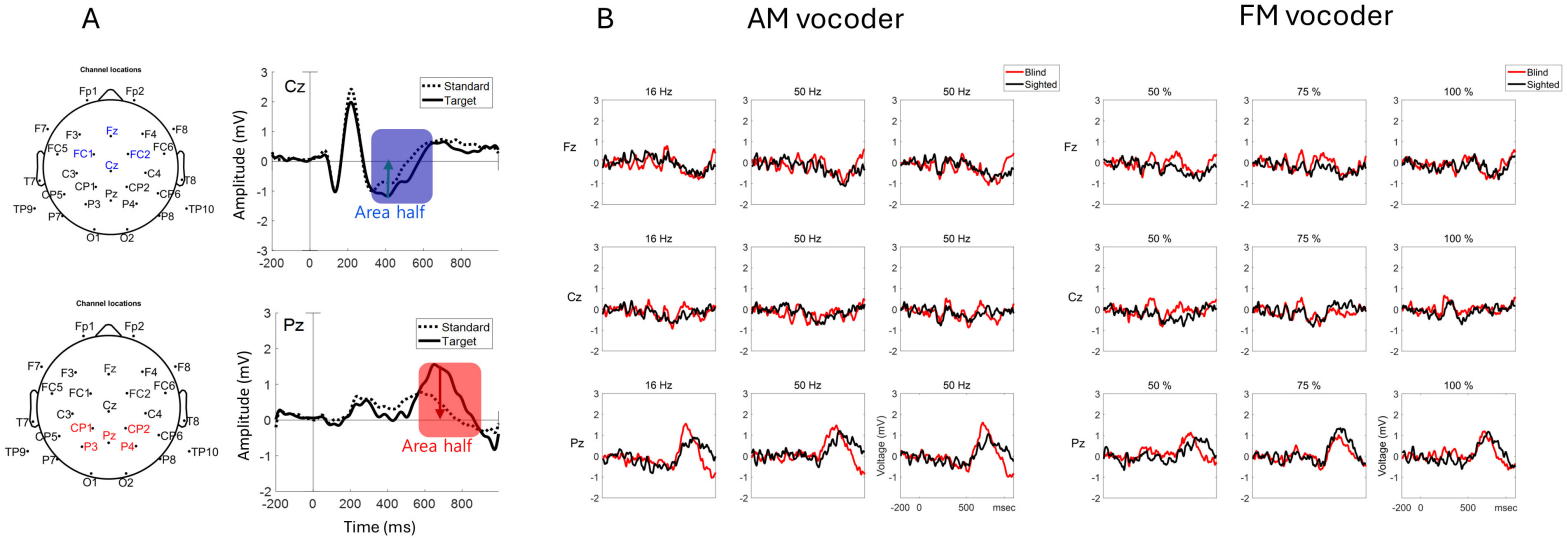
Sample waveforms of the N2 and P3b components **(A)** N2 was measured by averaging four frontocentral electrodes (Fz, FC1, FC2, and Cz) in the scalp map. P3b was measured by averaging five parietocentral electrodes (CP1, CP2, P3, P4, and Pz) in the scalp map. The blue shade represents the time window of the N2 component, and the red shade represents the P3b time window, computed from the grand average waveform of all subjects across all conditions. The blue and red arrows indicate the time point of each area's half. These representative waveforms were from Cz and Pz electrodes, shown for illustration. Both were collapsed from all conditions across all subjects. Difference waveforms of each condition **(B)**. Based on these difference waveforms, the time windows for amplitude modulation (AM) and frequency modulation (FM) were determined as 330–650 and 350–600 ms post-stimulus onset for N2, respectively and 560–895 and 565–895 ms post-onset for P3b, respectively. Positive values were plotted upward.

The data were preprocessed and analyzed with Brain Vision analyzer (version 2.0, Brain Products GmbH) and MATLAB R2019b (Mathworks) using EEGLAB v2021 (Delorme and Makeig, 2004) and Fieldtrip (Oostenveld et al., 2011) toolboxes. EEG was filtered with a high-pass filter at 0.1 Hz (Butterworth filter with a 12 dB/oct roll-off) and a low-pass filter at 50 Hz (Butterworth filter with a 24 dB/oct rolloff). Data were resampled at 256 Hz. Fast independent component analysis (Hyvärinen and Oja, 2000) was used to reject artifacts associated with eye blinks and body movement (average of 4 independent components, range 3–6) and reconstructed (Makeig et al., 1997), with transformation to the average reference. The EEG waveforms were time-locked to each stimulus onset and segmented from 200 ms before the stimulus onset to 1,000 ms after the stimulus onset. Baseline correction was then performed. The epochs with incorrect behavioral responses were excluded from further preprocessing. Before averaging, bad channels were interpolated using a spherical spline function (Perrin et al., 1989), and segments with values ±70 μV at any electrode were rejected. All of the subjects had data for at least 150–197 usable standard trials out of 210 trials and 63–87 usable target trials out of 90 trials. An average wave file was generated for each subject for each condition. According to previous studies', the latency ranges for N2 and P3b were determined based on the grand average computed across all conditions and participants. Accordingly, the N2 component in the current study was defined as the periods of 330–650 ms and 350–600 ms post-stimulus onset for AM and FM, respectively. The P3b component was defined as the periods of 560–895 ms and 565–895 ms post-stimulus onset for AM and FM, respectively. The peak latency and peak amplitude were measured by half-area quantification, which may be relatively unaffected by latency jitter (Luck, 2014; Finke et al., 2016). The ERP latency was quantified using the 50% area latency measure. We computed the signed area under the ERP waveform over a given latency range and then defined the time point that divides the area in half. This measure is known to be less affected by single-trial latency jitter and it is relatively insensitive to high-frequency noise (Petermann et al., 2009; Meyer et al., 2011; Luck, 2014). Difference waveforms were constructed by subtracting the target stimuli from the standard stimuli within each condition (Deacon et al., 1991). The area latency and amplitude of the N2 and P3b difference waveforms were compared between each condition and group. N2 was measured by pooling the signals from the frontocentral electrodes (Fz, FC1, FC2, and Cz), whereas P3b was measured by averaging the signals from the parietal electrodes (CP1, CP2, P3, P4, and Pz), as illustrated in Figure 3 and outlined in Finke et al. (2016).

### 2.4 Statistical analysis

Two-way repeated-measures analysis of variance (RM-ANOVA) was used to analyze the effects of group, AM vocoder, and FM vocoder on monosyllable recognition, as well as the latency and amplitude of the N2 and P3b components. *Post-hoc* paired *t*-tests, significance levels were set at 0.05 for multiple comparisons after applying Bonferroni's correction to the *p*-values. Pearson correlation analyses between AM or FM vocoded speech recognition and neural responses of the N2 and P3b components were performed with Bonferroni's correction (α = 0.05/6 = 0.008). All statistical analyses were performed using IBM SPSS software (ver. 25.0; IBM Corp, Armonk, NY, USA).

## 3 Results

### 3.1 Behavioral data

**Figure 4:**
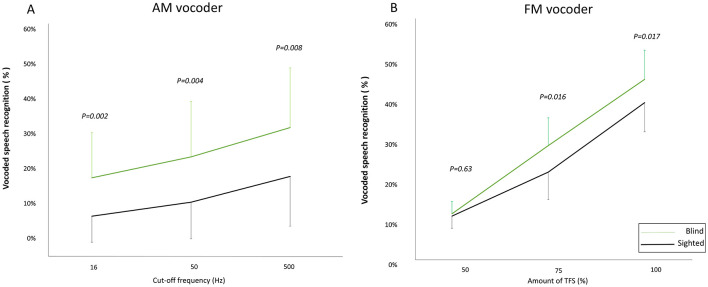
Vocoded speech recognition. Blind subjects (green line) show higher recognition of amplitude-modulated (AM) vocoded speech than sighted subjects (black line) at all envelope cutoff frequencies, with statistically significant differences (16 Hz: *p* = 0.002; 50 Hz: *p* = 0.004; 500 Hz: *p* = 0.008) **(A)**. Blind subjects (green line) show higher recognition rates of frequency-modulated (FM) vocoded speech than sighted subjects (black line), with significant differences at noise levels of 75% (*p* = 0.016) and 100% (*p* = 0.017). **(B)** Data points represent mean values, and error bars indicate standard deviations.

We measured the recognition of vocoded speech with the temporal envelope and TFS, each degraded at three different levels. A mixed two-way RM-ANOVA (two groups × envelope cutoff frequency) revealed significant main effects of group (*F*_(1,38)_ = 9.734, *p* = 0.003) and envelope cutoff frequency (*F*_(1.568,59.566)_ = 69.151, *p* < 0.001). However, there was no significant interaction between group and envelope cutoff frequency (*F*_(1.568,59.566)_ = 0.954, *p* = 0.372). *Post-hoc* tests using Bonferroni correction indicated that early-blind subjects outperformed sighted subjects in AM-vocoded speech recognition across all cutoff frequencies (16 Hz: *p* = 0.002; 50 Hz: *p* = 0.004; 500 Hz: *p* = 0.008; Table 2, Figure 4A).

Table 2Statistical summary of envelope cutoff frequency (amplitude modulated) vocoded speech.

**Table:** 

	**Sum of square**	***df***	**Mean square**	***F***	***p***	**η^2^_G_**
Cut-off frequency	211.517	1.568	134.936	69.151	<0.001^***^	0.645
Group	300.833	1	300.833	9.734	0.003^**^	0.204
AM^*^Group	2.917	1.568	1.861	0.954	0.372	0.024
Residual	116.233	59.566	1.951

**Table:** 

***Post-hoc***				
		**Mean difference**	**Standard error**	***p***
16 Hz	Ealy-blind vs. sighted	−2.75	0.842	0.002^**^
50 Hz	Ealy-blind vs. sighted	−3.25	1.065	0.004^**^
500 Hz	Ealy-blind vs. sighted	−3.50	1.246	0.008^**^

^**^p < 0.01, ^***^p < 0.001.

$\eta^{2}G=\frac{SSeffect}{SSeffect+SS\;subjects+SS\;error\;}$ (Small: < 0.01, Medium: 0.01~0.06, Large: 0.14).

For TFS, the RM-ANOVA (two groups × amount of TFS) showed significant main effects of group (*F*_(1,38)_ = 6.301, *p* = 0.016) and amount of TFS (*F*_(2,76)_ = 393.653, *p* < 0.001), and a significant interaction between these two factors (*F*_(2,76)_ = 4.363, *p* = 0.016). In the *post-hoc* tests using Bonferroni correction revealed that early-blind subjects showed better FM-vocoded speech recognition than sighted subjects, except at a TFS of 50% (50% TFS: *p* = 0.639; 75% TFS: *p* = 0.016; 100% TFS: *p* = 0.017; Table 3, Figure 4B).

Table 3Statistical summary of the amount of temporal fine structure (TFS; frequency modulated) vocoded speech.

**Table:** 

	**Sum of square**	***df***	**Mean square**	**F**	***p***	**η^2^_G_**
Amount of TFS	4,788.067	2	2,394.653	393.653	<0.001^***^	0.912
Group	140.833	1	140.833	6.301	0.016^*^	0.142
FM^*^Group	53.067	2	26.533	4.363	0.016^*^	0.103
Residual	462.200	76	6.082

**Table:** 

***Post-hoc***				
		**Mean difference**	**Standard error**	***p***
50%	Ealy-blind vs. sighted	−0.3	0.634	0.639
75%	Ealy-blind vs. sighted	−3.3	1.308	0.016^*^
100%	Ealy-blind vs. sighted	−2.9	0.057	0.017^*^

^*^p < 0.05, ^***^p < 0.001.

$\eta^{2}G=\frac{SSeffect}{SSeffect+SS\;subjects+SS\;error\;}$ (Small: < 0.01, Medium: 0.01~0.06, Large: 0.14).

Overall, the results indicate that early-blind subjects showed superior recognition compared with sighted subjects, even under conditions with degradation of the auditory temporal envelope and TFS. Speech recognition in early-blind subjects declined more with increasing TFS degradation, as evidenced by a significant interaction between group and the degree of TFS degradation. However, there was no difference between the groups regarding the impact of temporal envelope degradation on speech recognition.

### 3.2 EEG data

**Figure 5:**
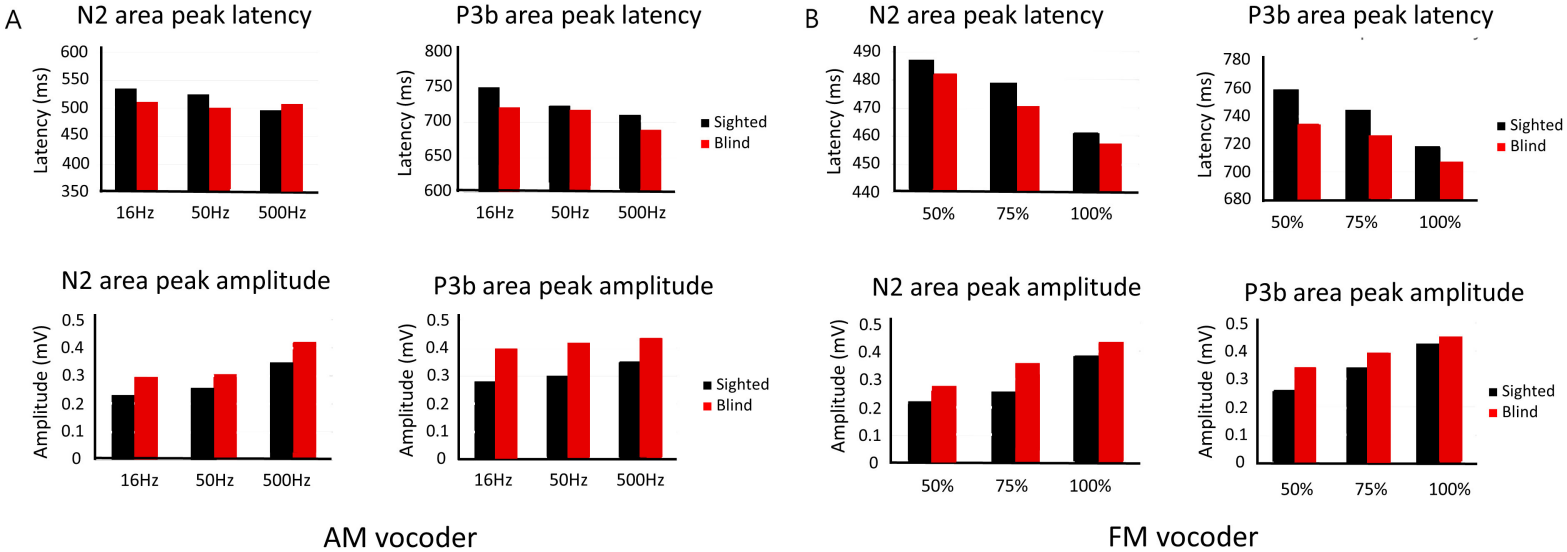
Mean latencies and amplitudes of N2 and P3b in the early-blind (red) and sighted (black) groups at amplitude-modulated conditions of 16, 50, and 500 Hz **(A)**, and frequency-modulated conditions of 50, 75, and 100% **(B)**.

The effect of envelope cutoff and group on the latency and amplitude of N2 and P3b was examined using mixed two-way RM-ANOVA (two groups × envelope cutoff frequency). The analysis revealed a significant effect of envelope cutoff frequency for the N2 amplitude (*F*_(1.549,58.881)_ = 7.244, *p* = 0.003) and P3b latency (*F*_(2,76)_ = 14.238, *p* < 0.001). The group effect for the P3b amplitude showed a trend toward significance (*F*_(1,38)_ = 4.081, *p* = 0.050), although the result did not reach the conventional threshold for statistical significance (*p* < 0.05; Table 4, Figure 5).

**Table 4:** Statistical summary of the effect of envelope cutoff frequency on the latency and amplitude of N2 and P3b components.

	**Sum of square**	***df***	**Mean square**	***F***	***p***	**η^2^_G_**
**N2**						
Latency	10,181.562	1.703	5,979.021	2.930	0.069	0.072
Group	4,940.833	1	4,940.833	1.363	0.250	0.035
latency^*^Group	8,167.917	1.703	4,796.527	2.351	0.111	0.058
Residual	1,32,047.396	64.709	2,040.619			
Amplitude	0.321	1.549	0.207	7.244	0.003^*^	0.160
Group	0.119	1	0.119	2.610	0.114	0.064
Amplitude^*^Group	0.002	1.549	0.002	0.055	0.908	0.001
Residual	1.683	58.881	0.029			
**P3b**						
Latency	25,494.200	2	12,747.100	14.238	<0.001^***^	0.273
Group	10,849.008	1	10,849.008	3.027	0.090	0.074
Latency^*^Group	3,030.067	2	1515.033	1.692	0.191	0.043
Residual	68,041.067	76	895.277			
Amplitude	0.056	1.520	0.037	1.752	0.180	0.044
Group	0.331	1	0.331	4.081	0.050	0.097
Amplitude^*^Group	0.008	1.520	0.005	0.238	0.727	0.006
Residual	1.205	57.759	0.021			

^*^p < 0.05, ^***^p < 0.001.

For TFS, the RM ANOVA (two groups × amount of TFS) showed a significant effect of the amount of TFS on N2 latency (*F*_(2,76)_ = 8.400, *p* < 0.001) and amplitude (*F*_(2,76)_ = 7.812, *p* < 0.001), as well as P3b latency (*F*_(2,76)_ = 8.734, *p* < 0.001) and amplitude (*F*_(2,76)_ = 15.868, *p* < 0.001). However, significant group effects were not found for the latency or amplitude of N2 or P3b (Table 5, Figure 5).

**Table 5:** Statistical summary of the effect of the amount of temporal fine structure on the latency and amplitude of N2 and P3b components.

	**Sum of square**	**df**	**Mean square**	***F***	***p***	**η^2^_G_**
**N2**						
Latency	13,051.926	2	6,525.963	8.400	<0.001^***^	0.181
Group	952.033	1	952.033	0.356	0.554	0.009
latency^*^Group	103.082	2	51.541	0.066	0.936	0.002
Residual	59043.367	76	776.886			
Amplitude	0.508	2	0.254	7.812	<.001^***^	0.171
Group	0.133	1	0.133	2.739	0.106	0.067
Amplitude^*^Group	0.019	1.922	0.010	0.287	0.751	0.007
Residual	2.469	76	0.032			
**P3b**						
Latency	24,050.317	2	12,025.158	8.734	<0.001^***^	0.187
Group	9,275.208	1	9,275.208	3.202	0.082	0.078
Latency^*^Group	967.117	2	483.558	0.351	0.705	0.009
Residual	1,04,633.900	76	1,376.762			
Amplitude	0.331	2	0.165	15.868	<0.001^***^	0.295
Group	0.081	1	0.081	2.014	0.164	0.050
Amplitude^*^Group	0.022	2	0.11	1.060	0.351	0.027
Residual	0.792	76	0.010			

^***^p < 0.001.

### 3.3 Correlation of neural response with behavioral data

**Figure 6:**
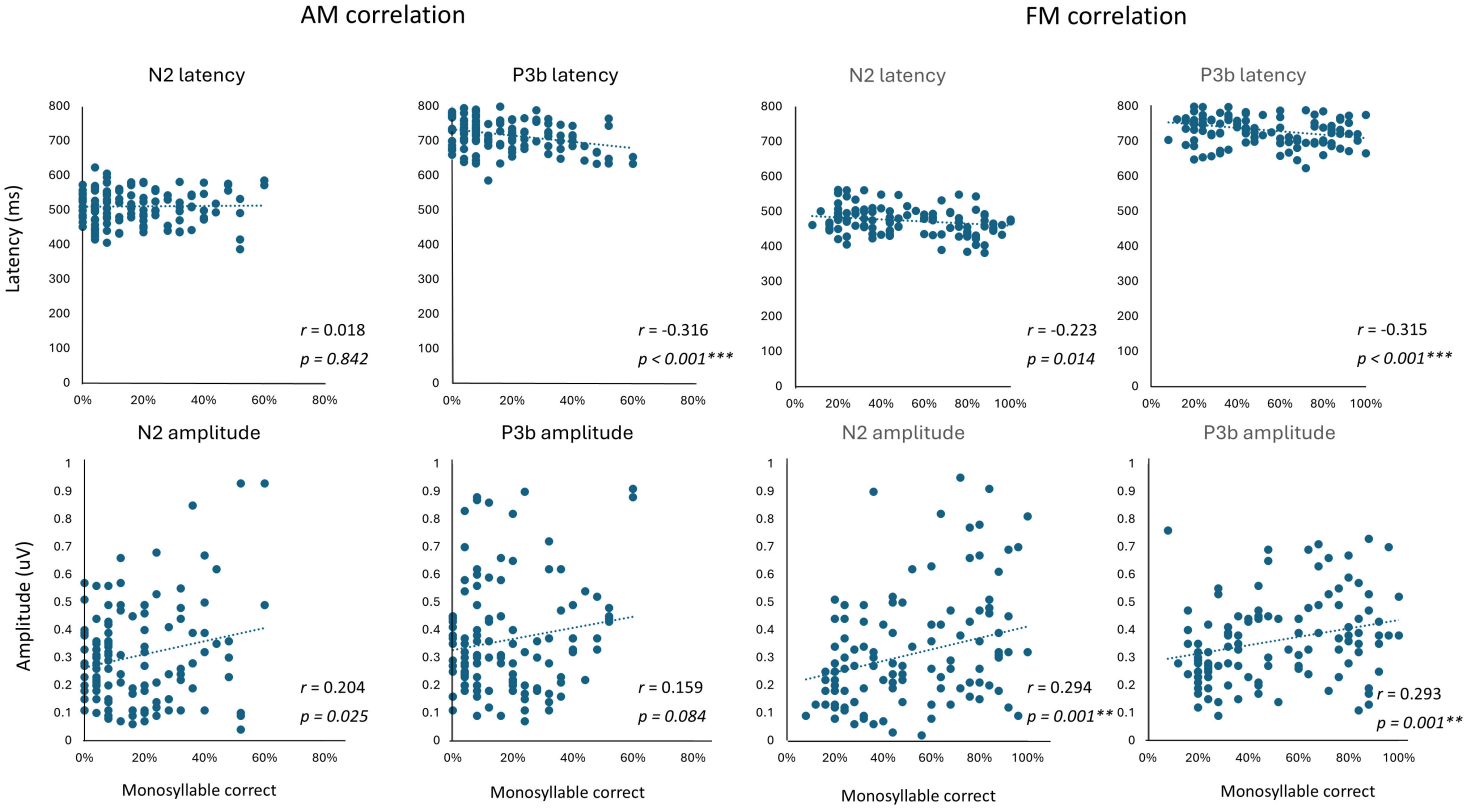
Correlations between AM- or FM-vocoded speech recognition and N2/P3b latency and amplitude. Scatter plots show the relationship between monosyllable recognition performance and the N2 and P3b components. Significant correlations after Bonferroni correction (*p* < 0.008) are indicated with asterisks.

We determined the correlations between AM or FM vocoded speech recognition and neural responses of the N2 and P3b components regarding latency and amplitude. A significant correlation was observed between the P3b latency and behavioral accuracy in AM vocoded speech recognition (*r* = −0.316, *p* < 0.001; Figure 6, left panel). Significant correlations were found between the N2 amplitude and behavioral accuracy in FM vocoded speech perception (*r* = 0.294, *p* = 0.001). Likewise, the P3b peak latency and amplitude exhibited significant correlations with behavioral accuracy (latency: *r* = −0.315, *p* < 0.001; amplitude: *r* = 0.293, *p* = 0.001; Figure 6, right panel).

## 4 Discussion

We investigated the effects of degraded temporal cues on speech recognition and semantic processing in early-blind individuals compared with sighted subjects. Our findings showed that early-blind participants demonstrated better speech recognition performance across almost all conditions, even with degradation of the temporal envelope and TFS, which is less detrimental for early-blind individuals. Furthermore, the P3b responses indicated that early-blind individuals may have enhanced cortical mechanisms for semantic processing in the case of degraded temporal cues. Supporting this notion, several studies have reported that early-blind individuals better utilize temporal cues compared with sighted individuals, including the processing of temporal-order judgment (Weaver and Stevens, 2006), temporal modulation detection (Shim et al., 2019), temporal patterns (Bae et al., 2022), and temporal resolution ability using gap detection (Muchnik et al., 1991). However, some studies found no difference in the gap detection threshold (Weaver and Stevens, 2006; Boas et al., 2011) and temporal bisection (Vercillo et al., 2016; Campus et al., 2019; Gori et al., 2020) between blind and sighted individuals. Several studies have demonstrated that early-blind participants were better at comprehending ultrafast speech (time-compressed speech) than sighted individuals, which underscores the adaptation of their auditory system to improve the encoding of temporal aspects of acoustic signals (Moos and Trouvain, 2007; Dietrich et al., 2013; Hertrich et al., 2013). Furthermore, both early- and late-blind individuals can acquire enhanced ability for ultrafast speech comprehension (Hertrich et al., 2013) and temporal modulation detection (Shim et al., 2019). Early-blind individuals prioritize temporal information in multidimensional selection tasks, initially selecting events based on timing rather than location, followed by a parallel selection incorporating both temporal and spatial attributes (Röder et al., 2007). The superior utilization of temporal cues in the brain by early-blind individuals compared with sighted individuals is presumed to be a result of compensatory plasticity due to long-term visual deprivation. Numerous neuroimaging studies have shown that blind individuals recruit the visual cortex to perform auditory functions (Leclerc et al., 2000; Gougoux et al., 2009; Collignon et al., 2011; Voss and Zatorre, 2012) and have a thicker visual cortex than sighted individuals (Voss and Zatorre, 2012). In addition, cross-modal plasticity occurs through the enhancement of pre-existing audiovisual connections (Beer et al., 2011; Collignon et al., 2013; Pelland et al., 2017) or the development of new audiovisual connections following the loss of vision (Karlen et al., 2006; Chabot et al., 2008). Synchronization of neuronal populations to the temporal dynamics of speech was observed in the primary visual cortex of early-blind individuals, along with functional connectivity between the temporal and occipital cortices (Van Ackeren et al., 2018). These findings suggest that the brain of blind individuals may adopt an architecture that enables them to track temporal cues, and the cerebrum appears to play a key role in temporal sound processing (Schulze and Langner, 1997; Eggermont, 2002; Bao et al., 2004).

The significant interaction between group and amount of TFS indicates that, while early-blind subjects may exhibit an overall advantage, the effectiveness of TFS shows a greater decrease with the level of degradation, thereby emphasizing the complexity of auditory processing in this population. In contrast, the impact of the envelope on speech recognition did not differ between the two groups, consistent with our previous results (Choi et al., 2024a). Earlier studies used two cutoff frequencies for the envelope cue (50 and 500 Hz), whereas the current study involved three cutoff frequencies (16, 50, and 500 Hz). However, the results were the same among the studies. The sensitivity of early-blind individuals to the reduction of TFS cues underlying the deterioration of speech recognition suggests that their ability to perceive speech in noise may be significantly compromised as they age or develop hearing loss. This is because the efficient use of TFS cues is severely limited with aging and hearing impairment (Lorenzi et al., 2006; Moore et al., 2006; Hopkins and Moore, 2007; Hopkins et al., 2008).

The EEG results provide further insights into the neural correlates of these behavioral findings. The significant effects of the envelope cutoff frequency on the N2 amplitude and P3b latency suggest that the degradation of temporal resolution influences higher-order cognitive processes involved in speech recognition and semantic integration. The amount of TFS showed significant main effects on the amplitude and latency of the N2 and P3b components. In the correlation analysis of neural responses with behavioral data, only one significant correlation was found for AM-vocoded speech, whereas three significant correlations were observed for FM-vocoded speech. This result might reflect a clearer effect of the condition, as observed in the RM ANOVA analysis of the latency and amplitude of N2 and P3b for FM-vocoded speech compared to AM-vocoded speech. The N2 component is associated with lexical information and semantic categorization (Schmitt et al., 2000; Van den Brink and Hagoort, 2004), whereas the P3b component is related to attention and updating working memory (Beynon et al., 2005; Henkin et al., 2015). The N2 and P3b results indicate that, with the degradation of the temporal envelope or TFS cues, there is an increased reliance on top-down processing for speech recognition. Similar patterns of N2/P3b utilizing the same speech oddball paradigm were observed in the context of degraded auditory spectral cues, which corresponded to reduced semantic integration with spectral degradation (Choi et al., 2024a,b). In adverse listening environments, the brain retrieves word meanings from our mental lexicon, which involves circuits for categorizing words based on their meanings. This process is reflected by a delayed latency and greater amplitude of the P3b component, which varies with the intensity of background noise (Henkin et al., 2008; Finke et al., 2016; Balkenhol et al., 2020). Other studies have also shown that individuals tend to depend more on top-down processing when spectral or temporal information is compromised or in the case of adverse listening conditions (Davis et al., 2005; Peelle and Davis, 2012).

The observed trend toward a significant difference in the P3b amplitude between the early-blind and sighted individuals hints at underlying differences in cognitive processing strategies between these groups, although this finding warrants further exploration with larger sample sizes. This finding could suggest that the brains of blind individuals may react more robustly to higher-order processing, including working memory. In a magnetoencephalography study, enhanced neural synchronization to acoustic fluctuations in early-blind individuals was observed in the theta range (corresponding to the syllabic rate) in the primary visual cortex (Van Ackeren et al., 2018). Furthermore, N2 and P3b were prolonged in cochlear implant users compared with subjects with nomal hearing, implicating a slower stimulus evaluation in the former, indicating slower access to lexical information and prolonged word evaluation. This finding highlights the impact of auditory processing on cognitive function (Henkin et al., 2008, 2015; Finke et al., 2016).

To our knowledge, this study is the first to compare speech recognition and relevant cortical-evoked potentials between early-blind and sighted individuals in listening environments involving degradation of the auditory temporal envelope and TFS. The results indicate that preserving TFS is crucial for speech recognition in visually impaired individuals with hearing impairment, thereby providing insights into the auditory rehabilitation of people with visual/auditory impairment. A limitation of this study is that we used vocoded speech to simulate degradation of the temporal envelope and TFS cues in young participants with normal hearing rather than in people with actual temporal resolution deficits. Future research should focus on elderly individuals with both visual and hearing impairments.

## Data Availability

The original contributions presented in the study are included in the article/supplementary material, further inquiries can be directed to the corresponding author.
